# Molecular models of the sperm head–tail coupling apparatus

**DOI:** 10.1242/jcs.264168

**Published:** 2025-10-08

**Authors:** Danielle B. Buglak, Brian J. Galletta, Nasser M. Rusan

**Affiliations:** Cell and Developmental Biology Center, National Heart Lung and Blood Institute, National Institutes of Health, Bethesda, MD 20892, USA

**Keywords:** Sperm, Head–tail connection, HTCA, LINC complex, Nuclear pore

## Abstract

A stable connection between the sperm head (nucleus) and tail (flagellum) is crucial for proper fertility. This linkage is mediated by centrioles, or their remnants, at a structure known as the head–tail coupling apparatus (HTCA). Although many proteins have been implicated at the HTCA, the precise molecular linkage that connects the head and tail is poorly understood. This Review proposes three molecular models for the HTCA based on the presence of three key components: nuclear envelope proteins, cytoplasmic proteins and centriole proteins. As it relates to these models, we discuss the current literature that describes the linkage from nuclear envelope proteins to cytosolic and centriole proteins, including a LINC-complex-based linkage, a nuclear pore complex linkage and a direct linkage that bypasses the outer nuclear membrane. Finally, we discuss outstanding questions in the field and how future studies might delineate the complex molecular machinery at the HTCA.

## Teaser

**Summary:** We propose three non-mutually exclusive models to describe sperm head–tail linkage crucial for sperm development and evading infertility, each with varying arrangements of nuclear envelope, cytoplasmic and centriole components.

## Introduction

**Fig. 1.**
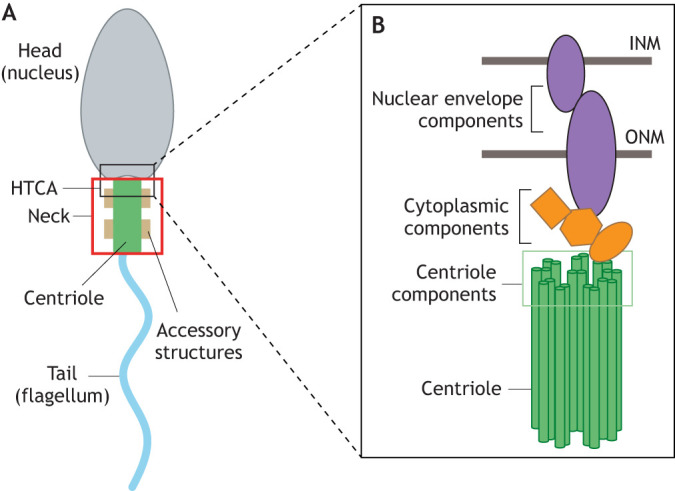
**Basic molecular model of the HTCA.** (A) Sperm are composed of a head (nucleus; gray) and tail (flagellum; blue) connected by a neck (red box) that includes the centriole (green) and accessory structures (brown). The molecular structure linking the nucleus and centriole is known as the head–tail coupling apparatus (HTCA; black box). (B) We propose that the HTCA must contain three key components: (1) NE components (purple), which reside in the INM and ONM to anchor the HTCA at the nucleus; (2) cytoplasmic components (orange) that act as a bridge between the nucleus and centriole; and (3) centriole components localized at the proximal end of the centriole (green).

Connections between nuclei and centrosomes are crucial in a variety of cellular contexts, including nuclear positioning within cells and centrosome separation during mitosis (reviewed in Bornens, 2008; Hannaford and Rusan, 2024; Vaughan and Dawe, 2011). A unique example of this connection is the ‘neck’ of sperm, which includes centrioles (the core structures of centrosomes) and accessory structures (Fig. 1A, red box). The neck attaches to sperm heads (nuclei) to facilitate the linkage between the head and tail (flagellum) (Fig. 1A, red box). Centrioles in this context are often referred to as ‘basal bodies’, but here we will only use the term ‘centriole’. The linkage between the neck and head relies on the head–tail coupling apparatus (HTCA; Gene Ontology term 0120212) (Fig. 1A, black box), a poorly understood molecular linkage between the neck and nucleus. The HTCA likely includes the centriole and accessory structures as well as cytoplasmic, nuclear envelope (NE) and intranuclear components, all of which form a stable connection that can withstand the forces of sperm swimming. Proper sperm head–tail linkage is essential for fertility. In humans, head–tail decoupling results in a condition known as acephalic spermatozoa syndrome. Individuals that are infertile show a high frequency of decoupling between the centrioles and the nucleus, suggesting a failure in proper HTCA assembly or function (Chemes et al., 1999; Chemes and Rawe, 2010; Mazaheri Moghaddam et al., 2021; Nie et al., 2022; Toyama et al., 2000; Wang et al., 2023; Zhu et al., 2016). These defects range from atypical head–tail linkage, due to HTCA mispositioning, to complete decapitation of the sperm head. Although there is some variation across species, electron microscopy (EM) analysis suggests that different HTCAs use similar accessory structures within the neck to mediate head–tail connections. However, the molecular components of the HTCA and their functions remain poorly understood. In this Review, we summarize the literature and hypothesize several compelling HTCA molecular models that warrant extensive future investigation.

Studies from both humans and mutant model organisms have implicated many proteins in the process of sperm head–tail connection (Anderson et al., 2009; Buglak et al., 2024; Deng et al., 2022; Galletta et al., 2020; Gan et al., 2024; Hall et al., 2013; Khanal et al., 2024; Kim et al., 2018; Kracklauer et al., 2010; Li et al., 2004, 2017a; Liska et al., 2009; Liu et al., 2018; Lu et al., 2021; Nie et al., 2022; Pasek et al., 2016; Sha et al., 2018; Shang et al., 2017, 2018; Shen et al., 2020; Sitaram et al., 2012; Texada et al., 2008; Tokuhiro et al., 2009; Wang et al., 2023; Xia et al., 2024; Yuan et al., 2015; Zhang et al., 2021a,b, 2024; Zheng et al., 2019; Zhu et al., 2018, 2016), but specific roles have been assigned to very few HTCA proteins (Table 1). It is often unclear whether head–tail connection failures seen in individuals with infertility or mutant model organisms are a consequence of HTCA failure or of errors earlier in sperm development, such as defects in chromosome segregation during meiosis, NE reformation or centriole structure. Furthermore, limitations of mouse genetics, such as the challenges associated with performing stage- and tissue-specific loss-of-function experiments and a lack of intact centrioles in mature rodent sperm, have made molecular studies of the HTCA in mammalian systems difficult. Although the precise molecular connections within the HTCA are still unclear, we can formulate models for the types of molecular components necessary to form a stable head–tail linkage. We place particular focus on studies in *Drosophila melanogaster*, where the molecular functions of some HTCA proteins have been studied in more depth. We discuss the key proteins known to influence both the establishment and maintenance of the HTCA, examine how these proteins might mechanistically fit into these models and address evidence that does not support elements of these models.

**Table 1.** Known, predicted and potential molecular components of the HTCA

Protein	Species	Localization	HTCA protein interactions	Function at the HTCA	References
**Nuclear envelope**					
*** Lamins***					
Lamin Dm0 (Lam; B-type lamin)	Flies	Developing manchette; undetectable in older spermatids	Unknown	Unknown	Fabbretti et al. (2016)
Lamin B1	Humans, rats, mice	HTCA	SUN5 by co-IP; SEPTIN12 by co-IP and yeast two-hybrid assay	Unknown	Elkhatib et al. (2015); Vester et al. (1993); Zhang et al. (2024)
Lamin B3	Humans, mice	HTCA (testis specific)	Unknown	Unknown	Elkhatib et al. (2015); Gob et al. (2010); Schutz et al. (2005)
*** LINC complex***					
Spag4	Flies	HTCA, manchette	Unknown	Required for head–tail linkage	Buglak et al. (2024); Kracklauer et al. (2010)
SUN5 (SPAG4L, TSARG4)	Humans, mice	HTCA	SEPTIN12, nesprin-3, lamin B1, Nup93 and CCDC113 (CFAP263) by co-IP; DNAJB13 and CENTLEIN by GST pulldown and co-IP	Required for head–tail linkage	He et al. (2024); Shang et al. (2017, 2018); Wu et al. (2024); Zhang et al. (2021a,b, 2024); Zhu et al. (2016)
SUN4 (SPAG4)	Mice	HTCA, manchette	SEPTIN12 by co-IP and yeast two-hybrid assay; SUN3 and nesprin-1 by co-IP	Involved in tightening lateral regions of the HTCA	Pasch et al. (2015); Yang et al. (2018); Yeh et al. (2015)
Nesprin-3	Mice	HTCA	SUN5 by co-IP	Not required for fertility	Ketema et al. (2013); Zhang et al. (2021b)
*** Nuclear pore complex***					
Nup358 (RANBP2)	Flies	Manchette	Mst27D by co-IP and affinity purification mass spectrometry	Links manchette to NE via Mst27D	Li et al. (2023)
Nup210L	Humans, mice	Unknown	Unknown	Potentially important for nuclear compaction and histone-to-protamine transition in humans, and sperm head and flagellar morphology in mice.	Al Dala Ali et al. (2024); Arafah et al. (2021); Walters et al. (2009)
Nup50 (NPAP60)	Rats	HTCA	Unknown	Unknown	Fan et al. (1997)
Nup93	Mice	Unknown	SUN5 by co-IP	Promotes nuclear mRNA export in spermatids	He et al. (2024)
NDC1 (TMEM48)	Mice	HTCA, developing manchette	SEPTIN12 by co-IP and yeast 2-hybrid assay	Important for meiotic progression	Akiyama et al. (2013); Lai et al. (2016); Yeh et al. (2015)
**Cytoplasm**					
* * ***Microtubule-related proteins***					
Hook1	Mice	Manchette, HTCA	Unknown	Required for linking manchette and flagellum to the nucleus and for proper positioning of microtubules in sperm	Mendoza-Lujambio et al. (2002)
DYNLT1 (tctex-1, Dlc90F)	Humans, mice, flies	Head, midpiece and tails in humans	Unknown	In flies, functions to bring nucleus and centriole together in early spermatids. Potentially involved in fertility in mice.	Caggese et al. (2001); Indu et al. (2015); Lader et al. (1989); Li et al. (2004); Sitaram et al. (2012)
Lis-1	Flies, mice	Developing manchette in flies	Asunder by co-IP	Functions with dynein to bring nucleus and centriole together in early spermatids in flies. Linked to infertility and spermatid differentiation in mice.	Nayernia et al. (2003); Sitaram et al. (2012)
Asunder	Flies	Nucleus	Lis-1 by co-IP	Recruits dynein to the NE to assist with centriole docking to the nucleus	Anderson et al. (2009); Sitaram et al. (2012)
Dynactin component p50 (Dynamitin, DCTN2-p50)	Flies	Developing manchette	Unknown	Knockdown reduces male fertility and mRNA levels of Lis-1, Spag4 and Yuri. Might function in dynein-mediated assembly of the HTCA.	Anderson et al. (2009); Sitaram et al. (2012); Wu et al. (2016)
Dynein heavy chain	Flies	Developing manchette	Unknown	Might function in dynein-mediated assembly of the HTCA	Anderson et al. (2009); Sitaram et al. (2012)
Mst27D	Flies	Manchette	Nup358 by co-IP and affinity purification mass spectrometry	Microtubule-binding protein that facilitates linkage of the manchette to the NE via Nup358	Li et al. (2023)
*** Chaperones***					
DNAJB13	Humans, mice	Axoneme, annulus, flagellum	SUN5 by GST pulldown and co-IP	Important for axoneme integrity	El Khouri et al. (2016); Guan et al. (2009); Guan and Yuan (2008); Shang et al. (2018)
BAG5	Mice	Manchette, HTCA	Spata6 and DYNLT1 by co-IP	Required for proper folding of Spata6 and dynein at the striated columns, thus regulating HTCA assembly.	Gan et al. (2024)
ODF1 (HSPB10)	Humans, mice	Manchette, tail	CCDC42 by co-IP	Organization of outer dense fibers and mitochondrial sheath; required for tight connection at the HTCA.	Hetherington et al. (2016); Hoyer-Fender (2022); Tapia Contreras and Hoyer-Fender (2019); Yang et al. (2012, 2014)
* * ***Other***					
Yuri gagarin	Flies	HTCA, manchette	Unknown	Specific to *Drosophila;* required for head–tail linkage and spermatid individualization.	Kracklauer et al. (2010); Texada et al. (2008, 2011)
SEPTIN12	Humans, mice	HTCA, manchette, annulus	SUN5 and γ-tubulin by co-IP; NDC1, lamin B1 and SUN4 by co-IP and yeast 2-hybrid assay	Important for head–tail linkage	Kuo et al. (2012, 2015); Lai et al. (2016); Lin et al. (2009, 2012); Shen et al. (2020); Yeh et al. (2015, 2019); Zhang et al. (2024)
PMFBP1 (STAP)	Humans, mice	HTCA	CENTLEIN by co-IP and GST pulldown	Required for head–tail linkage	Deng et al. (2022); Lu et al. (2021); Nie et al. (2022); Xia et al. (2024); Zhang et al. (2021a); Zhu et al. (2018)
Salto	Flies	Manchette, centriole adjunct, acrosome	Unknown	Might have a minor role in head–tail linkage	Augiere et al. (2019)
FAM46C (TENT5C)	Mice	Manchette	Unknown	Required for head–tail linkage	Zheng et al. (2019)
PRSS21 (TEST1, testisin)	Humans, mice	Cytoplasm, plasma membrane	Unknown	Tryptic serine protease important for head–tail linkage	Honda et al. (2002); Hooper et al. (1999); Netzel-Arnett et al. (2009); Scarman et al. (2001)
IFT88	Mice	HTCA, manchette, acrosome, tail	Unknown	Important at HTCA, likely due to a role in trafficking HTCA proteins along the manchette.	Kierszenbaum et al. (2011)
**Centriole**					
* * ***Centriole and PCM components***					
CENTLEIN	Mice	HTCA	SUN5 and PMFBP1 by co-IP and GST pulldown; CCDC113 by co-IP	Required for head–tail linkage	Wu et al. (2024); Zhang et al. (2021a)
Centrin1	Humans, mice	Centrioles	Unknown	Linked to male infertility and head–tail connection defects	Amargant et al. (2021); Avasthi et al. (2013); Fishman et al. (2018); Moretti et al. (2017, 2021, 2023)
CEP131, Dila	Mice, flies	Acrosome and HTCA	Unknown	Alignment and attachment of HTCA at later stages and organization of the axoneme and flagellum	Hall et al. (2013); Ma and Jarman (2011)
CEP135, Bld10	Humans, flies	Centriole in flies	Unknown	Involved in organization of the flagellum	Mottier-Pavie and Megraw (2009); Sha et al. (2017)
TSGA10	Humans, mice	Centriole, midpiece	ODF2 by yeast 2-hybrid assay	Involved in head–tail linkage and organization of the mitochondrial sheath	Behnam et al. (2015); Luo et al. (2021); Sha et al. (2018)
Centrobin	Rats, flies	Acroplaxome, manchette, HTCA and/or centrioles, flagellum in rats; centriole in flies	Unknown	Linked to the hypodactyly (*Hd*) locus in rats. Possibly involved in head–tail linkage and organization of the outer dense fibers and axoneme.	Liska et al. (2013, 2009); Moutier et al. (1973); Reina et al. (2018)
POC1	Humans, bulls, mice, flies	Atypical centrioles	Unknown	Anchors centrioles in later stages of spermiogenesis in flies; organization of outer dense fibers and flagellum in humans and mice.	Buglak et al. (2024); Fishman et al. (2018); Hua et al. (2023); Khanal et al. (2021); Khire et al. (2016); Platts et al. (2007); Turner et al. (2021)
CCDC42	Mice	HTCA, manchette	ODF1 by co-IP; ODF2 by affinity pulldown	Involved in head–tail linkage and organization of axonemal microtubules	Pasek et al. (2016); Tapia Contreras and Hoyer-Fender (2019)
CEP112	Humans, mice	Atypical centriole and axoneme	Unknown	Important for head–tail linkage in humans; facilitates translation of target mRNAs and might also contribute to integrity of the axoneme.	Sha et al. (2020); Zhang et al. (2024)
ODF2	Humans, mice	Centriole, manchette, flagellum	CCDC42 by affinity pulldown; TSGA10 by yeast 2-hybrid assay	Important for formation of outer dense fibers and a tight HTCA	Behnam et al. (2015); Ito et al. (2019); Tapia Contreras and Hoyer-Fender (2019); Zhu et al. (2022)
CCDC113 (CFAP263)	Mice	HTCA, manchette, flagellum	SUN5 and CENTLEIN by co-IP	Required for head–tail linkage and organization of the flagellum	Wu et al. (2024)
CCDC159	Mice	HTCA	Unknown	Required for head–tail linkage	Ge et al. (2024)
PLP	Flies	Centriole	Unknown	Restricts microtubule nucleation to the proximal end of the centriole to facilitate centriole docking at the NE	Galletta et al. (2020)
Spata6	Humans, mice, rabbits, rats, monkeys, fish	Striated columns, capitulum, manchette	BAG5 by co-IP	Required for head–tail linkage and organization of the axoneme, flagellum and outer dense fibers	Gan et al. (2024); Yuan et al. (2015)
Asl	Flies	Centriole adjunct	Unknown	Important for axoneme formation and possible attachment between the nucleus and centriole	Galletta et al. (2016); Khire et al. (2015, 2016)
Unc	Flies	Centriole adjunct	Unknown	Important for structure of the axoneme and male fertility	Baker et al. (2004)
**Other/unclassified**					
BRDT	Humans, mice	Unknown	Unknown	Important for chromatin remodeling, transcription and splicing in spermatocytes and spermatids; required for head–tail linkage.	Berkovits et al. (2012); Li et al. (2017a); Shang et al. (2007)
OAZ3	Mice	Unknown	Unknown	Required for tight head–tail connection	Tokuhiro et al. (2009)
SPATC1L	Humans, mice	HTCA	Unknown	Required for head–tail linkage; regulates PKA activity.	Kim et al. (2018)
SPATA20 (Ssp411)	Humans, mice, rats	Cytoplasm	Unknown	Important for head–tail linkage; regulation of Spata6.	Liu et al. (2018); Shi et al. (2004); Wang et al. (2023)

## The three key molecular components of the HTCA

**Fig. 2.**
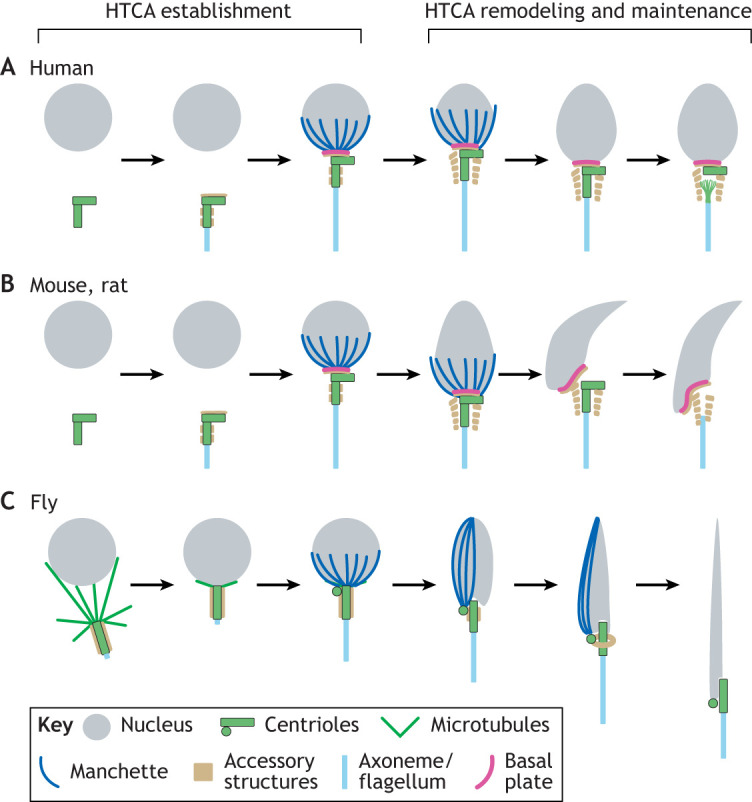
**Development of the HTCA.** HTCA development includes two phases: HTCA establishment (left) and HTCA remodeling and maintenance (right). Although the specifics of these processes can vary between species, the broad stages of HTCA development share many similarities. For simplicity, we have only illustrated structures relevant to our discussion of HTCA molecular mechanisms in this Review. (A) In humans, establishment involves the centrioles (green) making contact with the nucleus (gray). This contact is hypothesized to result from elongation of the axoneme and flagellum (light blue), which pushes against the cell membrane. The centriole then contacts a site of thickened NE known as the basal plate (pink). During establishment, accessory structures (tan), including the nine striated columns and the capitulum, begin to form, while the manchette microtubules (dark blue) begin to cover the caudal half of the nucleus. During remodeling and maintenance, the manchette continues to shape the nucleus while the striated columns and capitulum develop. Eventually, the manchette disappears, and the centriole that nucleates the axoneme is significantly reduced and remodeled into an atypical centriole, whereas the centriole contacting the basal plate remains intact. (B) In mice and rats, HTCA establishment is similar to that in humans. However, during HTCA remodeling and maintenance, both centrioles are degraded. (C) In flies, the initial contact between the nucleus and centriole during HTCA establishment is mediated by microtubules. An accessory structure called the centriole adjunct (tan) also arises at this time. During remodeling and maintenance, the manchette is lateralized to one side of the nucleus before disappearing. The centriole adjunct is significantly remodeled before disappearing in the mature sperm structure. The centriole that forms the axoneme, as well as an atypical centriole called the proximal centriole-like (PCL) structure, are both retained in mature *Drosophila* sperm.

Development of the HTCA begins following meiosis in early spermatids when contact between the nucleus and centriole is initiated; we term this stage HTCA establishment (Fig. 2) (Buglak et al., 2024; Dooher and Bennett, 1973; Fawcett, 1981; Fawcett and Phillips, 1969; Holstein and Roosen-Runge, 1981; Tates, 1971; Tokuyasu, 1975; Yeh et al., 2015). The precise mechanism driving HTCA establishment in mammals is not well understood, although flagellar growth is thought to push the centrioles towards the nuclear surface (Fig. 2A,B) (Fawcett and Phillips, 1969). However, this model is not supported by experimental evidence. In flies, establishment is driven by microtubules that bring the nucleus and centriole together (Fig. 2C) (Anderson et al., 2009; Galletta et al., 2020; Li et al., 2004; Sitaram et al., 2012). Once contact occurs, the HTCA rearranges and matures in a secondary process that we term HTCA remodeling and maintenance (Buglak et al., 2024). This phase includes the maturation of accessory structures, such as the mammalian capitulum and striated columns, and the *Drosophila* centriole adjunct, which are thought to be a form of modified pericentriolar material (PCM) (Fig. 2) (Dooher and Bennett, 1973; Fabian and Brill, 2012; Fawcett and Phillips, 1969; Riparbelli et al., 2020; Tates, 1971; Zamboni and Stefanini, 1971). The final HTCA architecture is evolutionarily diverse across species (Fig. 2), and it is possible that different organisms have evolved distinct molecular mechanisms for HTCA establishment and maintenance. Evolutionary convergence and divergence of the HTCA is an important area of investigation but will not be the focus of this Review. We will instead focus on common themes among species, all of which require a stable head–tail connection consisting of protein complexes that bridge the gap between the NE and the centriole (Fig. 1B). Some proteins that help form this bridge have been identified, whereas others are suggested based on their mutant phenotypes or their known functions in sperm and other cell types. Here, we divide HTCA proteins into three categories. First are NE components: some HTCA components must be integral to, or strongly associated with, the NE (Fig. 1B, purple). These components could potentially include inner nuclear membrane (INM) proteins but must include an outer nuclear membrane (ONM) protein to facilitate tail attachment. Second are cytoplasmic components: there is likely a protein complex (or complexes) in the cytoplasm that links the NE to the centriole (Fig. 1B, orange). Third are centriole components: some parts of the centriole must interact with the cytoplasmic or NE components to ensure that the tail remains anchored to the head (Fig. 1B, green). Although the specific protein interactions between these components might vary across species, the broad framework of NE, cytoplasmic and centriole components is likely conserved.

## Nuclear envelope components of the HTCA

**Fig. 3.**
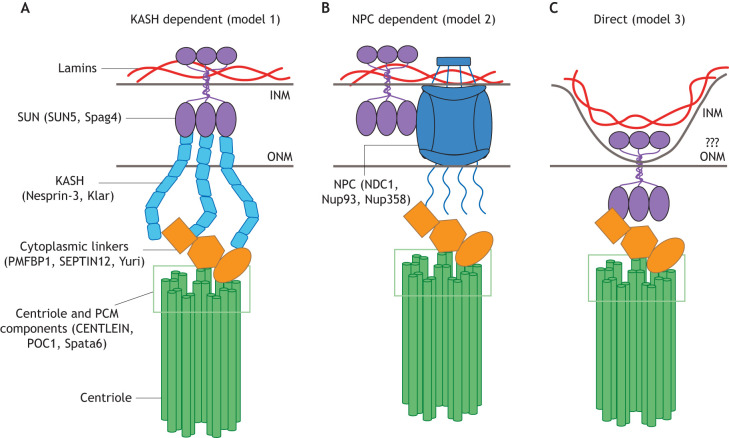
**Models for the NE components involved in HTCA linkage.** Spag4 in flies and SUN5 in mammals are known to be essential at the HTCA and are therefore included in all three potential models of NE linkage. For simplicity, we have depicted the HTCA with one centriole. However, it should be noted that different species have different centriole numbers and spatial organizations, which could impact their final molecular structures. (A) Model 1: HTCA linkage is KASH-domain protein-dependent, relying on a classical LINC complex between SUN proteins (purple) in the INM and KASH proteins (cyan) in the ONM to mediate the head–tail connection. KASH proteins then interface with cytoplasmic linkers (orange), such as PMFBP1, SEPTIN12 and Yuri, and centriole or PCM proteins (green box). (B) Model 2: HTCA linkage is NPC-dependent, relying on a physical connection between SUN proteins and the NPC (blue), which spans both nuclear membranes. Similar to the role of the KASH protein in model 1, the NPC would then interact with cytoplasmic and centrosomal proteins to link the head and tail. (C) Model 3: HTCA linkage is direct, with SUN proteins occupying the ONM, rather than their canonical position in the INM, or potentially distorting the INM into an unknown configuration. SUN proteins would then be available to directly interact with cytoplasmic and centrosomal proteins. This model presents a significant paradigm shift in the classical topology of SUN-domain proteins in the NE.

We propose three non-mutually exclusive models to describe the HTCA nuclear components (Fig. 3). Based on evidence from multiple species, all three models invoke a common component: a trans-INM Sad1/UNC-84 (SUN)-domain protein. The three models differ in the unique molecular linkages that traverse the ONM into the cytoplasm.

SUN-domain proteins are part of the linker of nucleoskeleton and cytoskeleton (LINC) complex, which connects the nuclear lamina to the cytoskeleton through the INM and ONM. Given that the HTCA links the nucleus and the centriole – a microtubule-based organelle – it is not surprising that SUN proteins (Fig. 3, purple) are essential for sperm formation in numerous species. In mammals, SUN1 and SUN2 are ubiquitously expressed, whereas SUN3, SUN4 (also known as SPAG4) and SUN5 are testis specific (Crisp et al., 2006; Frohnert et al., 2011; Gob et al., 2010; Jiang et al., 2011; Pasch et al., 2015; Shao et al., 1999; Tarnasky et al., 1998; Wang et al., 2006). Similarly, *Drosophila* have a ubiquitously expressed SUN protein, Klaroid (Koi), and a testis-specific SUN protein, Spag4 (Kracklauer et al., 2007, 2010). Spag4 localizes to the HTCA and along the side of the nucleus facing the manchette (known as the ‘dense complex’ in flies), another microtubule-based structure important for nuclear shaping and cargo transport along the elongating nucleus (Fig. 2, dark blue) (Anderson, 1967; Anderson et al., 1967; Clark, 1967; Fawcett et al., 1971; Kierszenbaum, 2002; Kierszenbaum et al., 2002; Tates, 1971). As the HTCA develops, Spag4 relocalizes on the nuclear membrane, forming a ‘cap’ that surrounds the centriole at the site of nucleus–centriole contact (Buglak et al., 2024; Kracklauer et al., 2010). Loss of Spag4 leads to male sterility in flies, with no mature sperm detectable in the seminal vesicles (Kracklauer et al., 2010). Fly *spag4* mutants exhibit impaired nucleus–centriole attachment that becomes more evident in mid-development spermatids, suggesting that Spag4 is essential for HTCA maintenance, not HTCA establishment.

In mice, SUN4 and SUN5 – the two orthologs of Spag4 – localize to the HTCA and appear to also play roles later in sperm development (Pasch et al., 2015; Shang et al., 2017; Yang et al., 2018; Yassine et al., 2015). SUN3, another testis-specific SUN protein, and SUN4 are primarily involved in nuclear shaping at the manchette (Gob et al., 2010; Pasch et al., 2015). Interestingly, SUN4 is dispensable for nucleus–centriole linkage (Pasch et al., 2015; Yang et al., 2018) but important for stabilizing the HTCA, particularly at regions lateral to the centrioles (Yang et al., 2018). Conversely, SUN5 is essential for nucleus–centriole linkage, as sperm from *Sun5* mutants are 98% headless. EM studies of *Sun5* mutant spermatids show clear spacing between the NE and centriole (Shang et al., 2017; Zhang et al., 2021b, 2024), which results in complete infertility. In fact, variants of human SUN5 account for ∼33–47% cases of acephalic spermatozoa syndrome (Shang et al., 2018; Zhang et al., 2021b; Zhu et al., 2016). Together, these studies highlight the importance of testis-specific SUN proteins in HTCA maintenance and fertility across the animal kingdom, a major factor as to why we consider SUN-domain proteins to be a common factor in all three nuclear linkage models presented below (Fig. 3).

### Model 1 – a traditional KASH-dependent LINC complex

LINC complexes span the NE to link the nuclear lamina with the cytoskeleton and are therefore excellent HTCA component candidates. Canonically, LINC complexes are composed of SUN-domain proteins residing in the INM, such as Spag4 and SUN5, which in turn interact with Klarsicht (Klar), ANC-1, Syne homology (KASH)-domain proteins located in the ONM extending into the cytoplasm (Haque et al., 2006; McGee et al., 2006; Padmakumar et al., 2005; Stewart-Hutchinson et al., 2008). Given their canonical role, LINC complexes could also be important in facilitating linkage between the nucleus and neck in spermatids (Fig. 3A, model 1).

A LINC complex at the HTCA would involve interactions between a SUN-domain protein such as Spag4 or SUN5 and KASH-domain proteins; however, a role for KASH proteins at the HTCA is yet to be identified, which could be due to several possibilities. First, KASH protein mutants used in past studies might not have been exhaustive. In this case, new null or mutant alleles that remove more than the KASH domain should be generated and tested to determine whether these proteins function at the HTCA independently of their KASH domains. It is also possible that KASH proteins function redundantly or that as-yet-undiscovered KASH proteins with roles in the HTCA exist. However, the possibility also remains that KASH-domain proteins are not involved in the HTCA (see models 2 and 3, below).

In *Drosophila*, deletion of the two known KASH-domain proteins, Klar and Msp-300, either individually or in combination, results in subfertility but does not disrupt other HTCA components (Kracklauer et al., 2010). In mice, the KASH protein nesprin-3 colocalizes and co-immunoprecipitates with SUN5 in testicular lysates, and *Sun5* mutant mice show disrupted nesprin-3 localization at the HTCA (Zhang et al., 2021b). This is consistent with a SUN5–nesprin-3 LINC complex functioning at the HTCA, as shown in model 1; however, loss of the KASH proteins nesprin-1, nesprin-2 or nesprin-3 does not lead to sterility in mice, suggesting an intact HTCA (Gob et al., 2010; Ketema et al., 2013; Zhang et al., 2007). In contrast, loss of the germline-specific KASH5 does result in infertility, but this is due to defects in earlier stages of meiosis (Horn et al., 2013).

Overall, the evidence for a single KASH-domain protein functioning at the HTCA is minimal. To test the classic LINC model at the HTCA, new alleles or knockdown tools for both mice and *Drosophila* will be required. For example, in *Drosophila*, mutant alleles of Klar and Msp-300 that eliminate more than the KASH domain could expose a KASH-domain-independent function. Given that KASH proteins are known to functionally compensate for one another (Banerjee et al., 2014; Rajgor et al., 2012), future experiments to test KASH protein redundancy via combinatorial loss of function will be important. However, such experiments are greatly complicated by the need for conditional, testes-specific mutations and temporally controlled protein knockdown systems due to the ubiquitous expression of KASH proteins and their important roles in other tissues. Development of such mutants and temporally controlled protein knockdown systems, such as deGradFP (Caussinus et al., 2011, 2013), are often more feasible in *Drosophila* than in mammalian systems.

Another exciting direction is identification of new KASH or KASH-like proteins. Immunoprecipitation followed by mass spectrometry and proximity labeling via biotin ligation are reasonable approaches. Another direction for identifying LINC components would be to dive deep into sequence data from individuals with infertility for genes that contain cryptic KASH domains; here, meta-analyses to cross-reference sperm proteomes with these data could prove fruitful. Finally, alternative hypotheses must be considered for how crucial SUN proteins like Spag4 and SUN5 molecularly link the sperm head and tail. We will cover two such possible models next.

### Model 2 – a nuclear pore complex-dependent linkage

We propose that a KASH-independent mechanism linking SUN proteins to cytoplasmic components of the HTCA could rely on nuclear pore complexes (NPCs) (Fig. 3B, model 2). Given that NPCs span the INM and ONM to facilitate protein transport, they could also form a trans-nuclear membrane structure important for HTCA assembly and fertility. Although a specific role for NPCs at the HTCA has not been described, there is increasing evidence that NPCs are important for normal fertility. A recent study has found that individuals with infertility have an altered distribution of NPCs in the sperm NE (Bragina et al., 2024). Another study has found that variants of the NPC protein Nup210L are associated with male infertility in humans, although the exact cause is unclear and could be a result of defective protein transport (Al Dala Ali et al., 2024; Arafah et al., 2021; Walters et al., 2009). Based on a preponderance of indirect evidence, we propose a speculative model that suggests a protein transport-independent role for NPCs during spermiogenesis.

Firstly, NPCs localize to the right place at the right time to facilitate a trans-nuclear membrane linkage at the HTCA. Several NPC proteins, including Nup50 and NDC1, localize to the HTCA in rodent spermatids (Fan et al., 1997; Lai et al., 2016), although whether they function in head–tail linkage is unclear. In both *Drosophila* and in mice, NPCs strikingly relocalize from the entire nuclear perimeter to cluster near the HTCA in the early stages of spermiogenesis (Fan et al., 1997; Lai et al., 2016; Li et al., 2023; Tates, 1971; Tokuyasu, 1974). It is unclear why the protein transport function of NPCs would require such relocalization, but we suggest this is likely structurally driven. Of note, EM studies in mice and bulls have found that NPCs relocalize towards the tail (though not explicitly at the HTCA) before incorporating into the redundant NE (RNE, a membrane structure of excess NE that is eventually discarded) (Ho, 2010; Tovich et al., 2004). Localization of NPCs to the RNE in late spermiogenesis does not necessarily exclude a role at the HTCA, as it is possible that NPCs function early in spermiogenesis in a transient role or that nucleoporins could function independently of the NPC at the HTCA. A more recent study utilizing electron cryo-tomography has shown that NPC architecture is significantly remodeled in mature human sperm, creating smaller pores with fewer structural elements (dos Santos et al., 2024 preprint). These modified NPCs exhibit significantly reduced nuclear transport, supporting our hypothesis that these proteins might now serve a different role at the HTCA.

Secondly, some evidence suggests that NPC proteins interact with SUN-domain proteins, both physically and functionally. SUN1 binds to the NPC protein Nup153 in HeLa cells (Li and Noegel, 2015; Li et al., 2017b), and numerous studies have identified a functional relationship between SUN1 and NPC proteins like Nup153, TPR and POM121 (Liu et al., 2007; Smith et al., 2022; Talamas and Hetzer, 2011; Zhou and Pante, 2010). The relationship between SUN proteins and NPCs in developing spermatids has not been extensively investigated, but a recent study has found that SUN5 co-immunoprecipitates with Nup93 (He et al., 2024), providing evidence for a plausible physical linkage between SUN proteins and NPCs at the HTCA (Fig. 3B).

Thirdly, there is evidence that NPCs link the nucleus and the manchette, which, like the centriole, is an extra-nuclear microtubule-based structure. In *Drosophila*, Nup358 is a component of the NPC cytoplasmic filaments and is likely linked to the manchette through interaction with the microtubule-binding protein Mst27D (Li et al., 2023). Whether the NPC facilitates manchette linkage in other species is less clear, but a study in mice has found that mutations in the RNA-binding protein ADAD1 result in both disruption of the manchette and dysregulation of NPCs (Potgieter et al., 2023), suggesting an intimate functional relationship between NPCs and the manchette in mammals. Given that many proteins are found at both the manchette and HTCA, including Spag4, SUN4, Yuri gagarin, IFT88 and CCDC42 (Buglak et al., 2024; Kierszenbaum et al., 2011; Kracklauer et al., 2010; Pasch et al., 2015; Tapia Contreras and Hoyer-Fender, 2019; Texada et al., 2008; Yang et al., 2018), and the striking relocalization of NPCs to the HTCA, it is reasonable to hypothesize that NPCs could serve as the missing bridge between SUN proteins and the centriole (Fig. 3B). Overall, the NPC is a promising area for future research in male infertility.

### Model 3 – direct binding of SUN proteins to cytoplasmic linkers

The third model we propose is that SUN proteins might directly interact with cytoplasmic linkers or centriole proteins (Fig. 3C, model 3). This unconventional model would require SUN proteins to span the ONM or engage an unknown membrane topology that renders the INM close to the ONM or even directly exposed to the cytoplasm. Surprisingly, localization of SUN proteins outside the INM is not without precedent. SUN1η, a testis-specific isoform of SUN1, localizes outside of the nucleus near the acrosome (a Golgi-derived organelle at the rostral end of the nucleus that contains important enzymes for fertilization) as part of the anterior acrosomal membrane system (Gob et al., 2010). SUN5 itself has also been suggested to localize to the ONM in nuclear membrane topology assays and directly interacts with a portion of the centrosome protein CENTLEIN (also known as CNTLN) in immunoprecipitation experiments using purified recombinant proteins (Zhang et al., 2021a). SUN4 has also been found to localize outside of the nucleus in the outer dense fibers of the sperm neck in mice (Shao et al., 1999), although this could not be replicated in a later study (Pasch et al., 2015). Thus, it is reasonable to hypothesize that SUN-domain proteins might bypass other nuclear membrane linker proteins and directly interact with HTCA cytoplasmic or centriole components.

A similar interaction has been suggested in *Drosophila*. The *Drosophila*-specific cytosolic gravitaxis (directional movement in response to gravity) protein Yuri shares similar localization to Spag4 (Buglak et al., 2024; Kracklauer et al., 2010) and *yuri* mutants have a nucleus–centriole decoupling phenotype identical to that of *spag4* mutants (Kracklauer et al., 2010; Texada et al., 2008). Although these data are consistent with Spag4 and Yuri closely functioning together, there is no evidence of Spag4 in the ONM and it is unclear if or how Spag4 might directly interact with cytosolic Yuri. Taken together, evidence is mounting that testis-specific SUN proteins like Spag4 and SUN5 have unique properties and localizations, including non-canonical membrane localization and membrane-independent localization. We argue that these findings should remind the field that the breadth of their functions might be missed by rigid adherence to dogmatic views of the LINC complex.

## Cytoplasmic components of the HTCA

EM studies of the HTCA indicate that the space between the centriole and NE varies between species, suggesting that a range of molecular components could be required to bridge the cytoplasmic gap (Fig. 1B, orange). In this section, we examine the proteins implicated in this role and how they might function as part of the proposed nuclear component models.

### The microtubule motor dynein

Microtubules and their associated motors play a wide range of essential functions. In sperm, microtubules are the main structural components of the tail but are also important for nucleus–centriole attachment. In *Drosophila*, the microtubule motor dynein captures microtubules nucleated at the nuclear surface from the proximal end of the centriole to bring the two organelles together (Anderson et al., 2009; Galletta et al., 2020; Li et al., 2004; Sitaram et al., 2012). Studies in mammalian sperm cells that test a role for microtubules and dynein in nucleus–centriole attachment have not been reported. Instead, the longstanding model in mammals suggests that flagellum elongation pushes the centrioles towards the nuclear surface (Fawcett and Phillips, 1969).

Dynein has several important functions in sperm. ‘Axonemal dynein’ is part of the axoneme, a core microtubule-based structure that powers flagellar beating. Numerous studies from individuals with infertility indicate that axonemal dynein has an integral role in sperm motility (reviewed by Levkova et al., 2022). The roles of ‘cytoplasmic dynein’ and its co-factor dynactin in mammalian spermiogenesis are less clear. However, the dynein light chain subunit DYNLT1 [also known as tctex-1 or dynein light chain 90F (Dlc90F) in flies] of both cytoplasmic and axonemal dynein is highly expressed in testes and might be linked to sterility in mice (Lader et al., 1989). In humans, DYNLT1 localizes to the head, midpiece and tail of mature sperm, and reduced DYNLT1 expression is associated with infertility (Indu et al., 2015). How DYNLT1 specifically functions in mammalian sperm has not been tested.

Studies from *Drosophila* clearly indicate an important role for the dynein–dynactin motor complex. Mutants of *tctex-1* have defects in nucleus–centriole attachment and are completely sterile despite normal flagellar elongation and axoneme formation (Caggese et al., 2001; Li et al., 2004; Sitaram et al., 2012). Similarly, mutations in the dynein adaptor *lis-1* or the dynein regulator *asunder* (*asun*) impair nucleus–centriole attachment during early stages of spermiogenesis, leading to male sterility (Anderson et al., 2009; Sitaram et al., 2012).

Consistent with these mutant phenotypes and a role in nucleus–centriole attachment, dynein, the p50 subunit of dynactin (also known as DCTN2-p50) and the dynein adaptor Lis-1 all localize at the surface of the NE in early spermatids (Anderson et al., 2009; Li et al., 2004; Sitaram et al., 2012). Once dynein and Lis-1 have accumulated at the NE, they capture microtubules that emanate from the proximal end of the centriole to bring the centriole to the nuclear surface, initiating HTCA formation (Galletta et al., 2020). How dynein is anchored at the nucleus is not fully understood but is key to understanding how dynein functions at the nucleus and within the HTCA. In other cellular contexts – and in agreement with our HTCA models (Fig. 3) – dynein can be recruited by both LINC complexes and NPCs (Bolhy et al., 2011; Fridolfsson et al., 2010; Garner et al., 2023; Horn et al., 2013; Malone et al., 2003; Splinter et al., 2010). Thus, we hypothesize a direct linkage between dynein and a SUN-domain, KASH-domain or NPC protein.

Dynein is clearly essential for the early stages of HTCA development, but whether it functions at the HTCA in later stages is unknown. This is difficult to test due to the severe phenotypes in early spermiogenesis associated with dynein dysfunction. To effectively test this, the obstacle of specifically manipulating dynein in later stages of spermiogenesis, after the initial HTCA has already been assembled, must be overcome.

### Yuri gagarin

Yuri is a *Drosophila*-specific gravitaxis protein that plays a crucial role at the head–tail connection during spermiogenesis. Yuri has multiple isoforms that show ubiquitous expression, including a short 30 kDa isoform, multiple 64–65 kDa isoforms and multiple 101–107 kDa isoforms (Texada et al., 2008). During spermiogenesis, Yuri first localizes to the developing manchette before coalescing at the HTCA to surround the centriole at its point of contact with the nucleus (Buglak et al., 2024; Kracklauer et al., 2010). Localization of Yuri to the manchette is dependent on dynein and Spag4 (Kracklauer et al., 2010; Texada et al., 2008). During later stages of spermiogenesis, Yuri recruits tropomyosin 1 to specialized F-actin cones that are important in the formation of mature sperm (Texada et al., 2011). *YuriF64* mutants lead to loss of the 65 kDa isoforms in all tissues and specific loss of the 30 kDa isoform in the testis. Though viable, male *YuriF64* mutants are completely sterile, with sperm exhibiting significant head–tail detachment (Kracklauer et al., 2010; Texada et al., 2008). This phenotype is identical to that of *spag4* mutants, and Yuri and Spag4 colocalize throughout spermiogenesis, suggesting that they function in the same pathway (Buglak et al., 2024; Kracklauer et al., 2010; Texada et al., 2008). Furthermore, localization of Yuri to the manchette is Spag4 dependent, and Spag4 and Yuri show co-dependent localization to the HTCA (Kracklauer et al., 2010).

If and how Spag4 and Yuri molecularly associate at the HTCA is unclear, as no direct interaction has been described and because they are likely in different cellular compartments. Yuri does not contain a KASH domain, transmembrane domain or any putative nuclear localization signals, suggesting that it is a cytoplasmic protein rather than an NE protein. Interaction between Yuri and tropomyosin 1 further emphasizes cytoplasmic localization of Yuri (Texada et al., 2011). In both Spag4 and dynein light chain mutants, Yuri does not localize to the HTCA and instead localizes ectopically along the centriole (Kracklauer et al., 2010; Texada et al., 2008), further suggesting that Yuri is not bound to the NE. Thus, we favor a model where an ONM protein, such as a KASH protein or NPC, links Yuri to Spag4 rather than a model involving a direct Yuri–Spag4 interaction (Fig. 3A,B; models 1 and 2). These data also raise the possibility that Yuri interacts with one or more centriole proteins and could facilitate the interaction between the centriole and NE. Although Yuri is a non-conserved protein, it is plausible that an unknown functional ortholog is present in mammalian sperm. Such an ortholog could be identified by cross-referencing proteins linked to fertility defects with tropomyosin interactors in mammalian sperm, or with proteins that show structural similarities with Yuri, as predicted by programs such as AlphaFold (Jumper et al., 2021).

### Septins

Septins are GTP-binding proteins that oligomerize to form cytoskeletal structures, including filaments and rings, that are important in processes like cell division and polarity (reviewed by Bridges and Gladfelter, 2015). Testis-specific SEPTIN12 is expressed post-meiotically in mouse spermatids (Lin et al., 2009) and localizes to several structures in sperm, including at the head–tail connection in spermatids (Lin et al., 2009, 2012; Shen et al., 2020). Several studies have identified SEPTIN12 variants in individuals with infertility with sperm morphology defects (Kuo et al., 2012; Lin et al., 2012). These individuals frequently present with alterations of the sperm tail, although SEPTIN12 variants can also result in defects in the sperm neck (Kuo et al., 2012, 2015). SEPTIN12 mutant mice show a significant increase in acephalic spermatozoa with either incomplete or total loss of the striated columns and capitulum (Shen et al., 2020). This suggests that SEPTIN12 is important during HTCA development and assembly, though precisely what role it plays during neck development is unclear. Interestingly, SEPTIN12 interacts with SUN4 and co-immunoprecipitates with SUN5 in spermatids (Yeh et al., 2015, 2019; Zhang et al., 2024). Furthermore, SUN5 knockout mice show loss of SEPTIN12 from the sperm neck, and SEPTIN12 overexpression in human cell lines causes SUN4 and SUN5 to mislocalize from the NE into aggregates (Yeh et al., 2019; Zhang et al., 2024). Together, these findings suggest that localization of SEPTIN12 and SUN4 or SUN5 to the HTCA are co-dependent and that these proteins might form a complex to link the head and tail. However, although variant forms of SEPTIN12 do not properly assemble key HTCA structures (Shen et al., 2020), spermatids expressing variants of SUN4 and SUN5 exhibit normal HTCA assembly (Pasch et al., 2015; Shang et al., 2017; Yang et al., 2018), suggesting that SEPTIN12 might have functions in HTCA assembly independent of SUN4 and SUN5. Furthermore, co-immunoprecipitation (co-IP) experiments using SEPTIN12-overexpressing cultured testicular carcinoma cells have found that SEPTIN12 pulls down γ-tubulin (Yeh et al., 2019). We therefore favor a model where SEPTIN12 serves as a cytoplasmic linker. SUN5 interacts with SEPTIN12 in co-IP experiments (Zhang et al., 2024), but direct binding has not been tested. Interestingly, SEPTIN12 also forms a complex with the NPC protein NDC1 (Lai et al., 2016), suggesting that SEPTIN12 could function at the HTCA alongside the NPC, consistent with our model 2 (Fig. 3B). In addition, other septins, including SEPTIN1, SEPTIN2, SEPTIN10 and SEPTIN11, localize to the HTCA, where they likely hetero-oligomerize (Shen et al., 2020). Whether or not variants of these septin proteins similarly result in infertility and head–tail connection defects is unknown.

### PMFBP1

Polyamine-modulated factor 1-binding protein 1 (PMFBP1, also called STAP) is a testis-specific protein that localizes to the HTCA (Ohuchi et al., 2001; Zhu et al., 2018). PMFBP1 primarily consists of coiled-coil domains, and its precise molecular function is not well described. It is primarily thought to be a scaffolding protein, though a more recent study has found that PMFBP1 can interact with histone deacetylases and might regulate flagellar assembly (Xu et al., 2023). Variants of PMFBP1 and SUN5 account for 70% of cases of acephalic spermatozoa syndrome in humans (Deng et al., 2022; Lu et al., 2021; Nie et al., 2022; Xia et al., 2024; Zhang et al., 2021a; Zhu et al., 2018), clearly placing PMFBP1 as a crucial HTCA element. Loss of PMFBP1 presents a phenotype identical to loss of SUN5: sperm heads and tails become detached between the nucleus and basal plate. Although PMFBP1 and SUN5 have not been found to directly interact (Zhang et al., 2021a), their shared localization and variant phenotypes suggest that they function together to link the sperm head and tail. A centrosome protein, CENTLEIN (discussed below), localizes between PMFBP1 and SUN5 at the HTCA, and a portion of CENTLEIN directly interacts with both SUN5 and PMFBP1 in pulldown experiments using purified recombinant proteins (Zhang et al., 2021a). The precise localization of PMFBP1 within the HTCA is unknown, although we propose it is a cytoplasmic linker. Due to the severity of HTCA phenotypes observed in SUN5, PMFBP1 and CENTLEIN mutants, this complex is likely the most essential and well-understood linkage between the nucleus and centriole, but counterparts to PMFBP1 and CENTLEIN in other species have not yet been identified, limiting the genetic approaches available to investigate this complex further.

## Centriole components of the HTCA

Either centrioles or remnants of deconstructed centrioles are present in the neck of sperm in all studied organisms in the animal kingdom; therefore, centriole proteins are included in our proposed models (Fig. 1B, green). Evidence suggests that centriole proteins interface with cytoplasmic linkers as well as directly with NE proteins, indicating a complex molecular topology at the HTCA.

### CENTLEIN

As mentioned above, CENTLEIN is a centrosome-associated protein that is important in the head–tail linkage. CENTLEIN was first described as a regulator of centrosome adhesion in human cell lines (Fang et al., 2014). In 2021, CENTLEIN was identified in a small-scale screen to identify SUN5 interactors in sperm (Zhang et al., 2021a), which found that CENTLEIN interacts with both SUN5 and PMFBP1. Because SUN5 (a INM protein) and PMFBP1 (likely a cytoplasmic protein) do not directly bind, CENTLEIN has been proposed to link SUN5 and PMFBP1 at the HTCA. In support of this model, CENTLEIN loss-of-function mutants share an identical phenotype with SUN5 and PMFBP1 mutants (Zhang et al., 2021a). Interestingly, CENTLEIN can localize directly to microtubules when overexpressed in HeLa and U2OS cells, suggesting a centriole-independent function (Jing et al., 2016). Alternatively, centriole-bound CENTLEIN could mediate interactions in a more intricate complex that includes the NE protein, the cytoplasmic linker and proteins on the centriole.

Another gap in our understanding of CENTLEIN, which is not a transmembrane protein, is how it can directly interact with the INM protein SUN5. This is very similar to the unresolved relationship between the *Drosophila* HTCA protein Spag4 and the cytoplasmic protein Yuri. Interestingly, some evidence indicates that SUN5 is not embedded in the INM in the canonical way (with its C-terminal SUN domain in the perinuclear space and its N terminus in the nucleoplasm, like a typical SUN protein), which would isolate it from the cytoplasm. Instead, SUN5 might be exposed to the cytosol, allowing direct interaction with CENTLEIN (Zhang et al., 2021a). This is consistent with our model 3 and serves as further evidence that testis-specific SUN-domain proteins might have unique properties (Fig. 3C). More work is needed to understand the topology of the NE and the nature of the SUN5–CENTLEIN–PMFBP1 complex, as many crucial details remain unknown.

### POC1

POC1A and POC1B are centriole proteins involved in centriole integrity and length control (Fourrage et al., 2010; Pearson et al., 2009; Venoux et al., 2013). Fly *poc1* mutants are sterile (Khire et al., 2016), and a variant of *POC1B* leading to infertility has recently been discovered in humans and mice (Hua et al., 2023). Furthermore, some individuals with infertility have altered levels of POC1B at the centrioles and reduced *POC1B* mRNA in their sperm (Platts et al., 2007; Turner et al., 2021). In flies, Poc1 is required for development of the proximal centriole-like (PCL) structure (Blachon et al., 2009; Jo et al., 2019), which is a crucial centriolar structure positioned adjacent to the centriole and immediately caudal to the sperm nucleus. Initial studies found that Poc1 in sperm is required for centrosome formation following fertilization and for early zygotic divisions (Khire et al., 2016). More recent work has identified a novel role for Poc1 and the PCL in stabilizing the head–tail connection (Buglak et al., 2024). Centrioles in *poc1* mutants lose their tight association with the nucleus and often become detached in the final stages of spermiogenesis (Buglak et al., 2024). How Poc1 stabilizes the HTCA is unclear, although based on its localization directly adjacent to the nucleus in fly spermatids, Poc1 and the PCL might directly interact with an as-yet-unidentified NE protein to stabilize the HTCA. Whether the POC1 proteins facilitate either development or remodeling of sperm centriole structures in mammals remains unknown.

### Spata6

Spata6 is a sperm protein that localizes to the neck and is highly conserved in vertebrates, being found in humans, monkeys, mice, rats, rabbits and fish (Yuan et al., 2015). Specifically, Spata6 is part of the striated columns and capitulum at the HTCA, the modified PCM that surrounds the centrioles and axoneme. Although Spata6 protein exclusively localizes to the neck in spermatids, its mRNA is expressed in both earlier spermatocytes and later spermatids (Oh et al., 2003; Yuan et al., 2015). Spata6 mutations are linked to acephalic spermatozoa and male infertility in mice, though it is unknown whether similar variant forms of Spata6 lead to infertility in humans (Yuan et al., 2015). Mice lacking Spata6 fail to form the striated columns and capitulum, resulting in breakage between the head and tail. Evidence suggests that Spata6 functions with the SUN5–CENTLEIN–PMFBP1 complex, as Spata6 localization to the HTCA is dependent on SUN5 and PMFBP1 (Zhu et al., 2018). Thus, a linkage between SUN5–CENTLEIN–PMFBP1 and Spata6 could connect the nucleus to the capitulum in the neck. Whether Spata6 interacts with other centriole proteins to link the striated columns and capitulum directly with the centriole is unknown.

## Nuclear attachment of centrioles and centrosomes through specialized LINC complexes

Aside from attachment at the neck in spermiogenesis, there are other contexts in which centrioles and centrosomes form attachments to the NE that could provide insight into the molecular composition of the HTCA. Many connections between nuclei and centrioles are dependent on classical LINC complexes (Chang et al., 2015, 2019; Hale et al., 2008; Infante et al., 2018; Obino et al., 2016; Roux et al., 2009; Salpingidou et al., 2007; Schneider et al., 2011; Zhang et al., 2009). However, given that there is little evidence for KASH proteins functioning at the HTCA, other specialized LINC complexes might provide insight into the nucleus-centriole linkage at the HTCA.

**Fig. 4.**
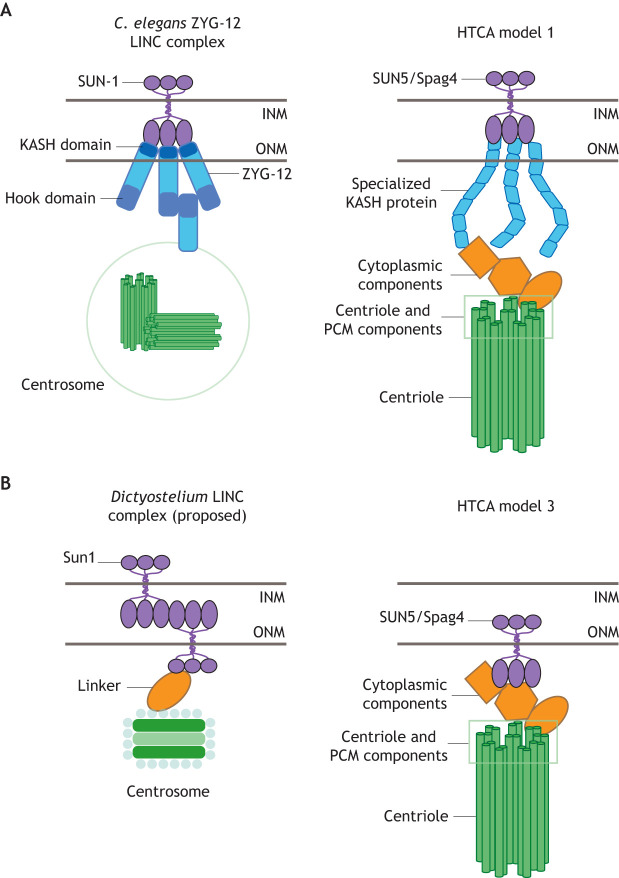
**Specialized LINC complexes in other systems provide clues to the molecular linkage at the HTCA.** (A) In *C. elegans*, the specialized LINC complex (shown to the left) formed by SUN-1 (purple) and ZYG-12 (cyan) anchors the centrosome (green) to the nucleus. One isoform of ZYG-12 contains a Hook domain exposed to the cytoplasm and a KASH domain that binds to SUN-1 in the INM. A second isoform of ZYG-12 that lacks the KASH domain facilitates the linkage of this unique LINC complex to the centrosome. This connection is reminiscent of the proposed KASH-dependent HTCA model 1 (shown to the right), which might rely on specialized KASH proteins to bridge the gap between SUN5/Spag4 and the centriole. (B) In *Dictyostelium*, a unique LINC complex (shown to the left) formed by Sun1 (purple) embedded in both the INM and ONM has been suggested to link the nucleus and centrosome, potentially through an unknown cytoplasmic linker protein (orange). This is reminiscent of the proposed HTCA model 3 (shown to the right), where SUN5/Spag4 occupies the ONM to directly interface with cytoplasmic and centriolar proteins.

In *Caenorhabditis elegans*, a unique LINC complex connects the nucleus and centriole independently of microtubules (and actin). The *C. elegans* KASH protein ZYG-12 (Malone et al., 2003; Minn et al., 2009), which contains a transmembrane domain, interacts with and is dependent on SUN-1 for its localization to the NE. ZYG-12 is unusual in that it is also a member of the Hook family of microtubule-binding proteins, which do not typically contain a transmembrane domain (Kramer and Phistry, 1999; Walenta et al., 2001). A KASH domain-less ZYG-12 isoform localizes directly to the centrosome, and this localization is dependent on dynein. Thus, linkage between the NE and centriole in *C. elegans* occurs through interactions between SUN-1 in the INM with the KASH domain-containing isoform of ZYG-12 in the ONM. This ZYG-12 then dimerizes with the KASH domain-less, cytosolic ZYG-12 bound to the centrosome. Given that the known KASH-domain proteins do not seem to significantly impact fertility in other species (Ketema et al., 2013; Kracklauer et al., 2010; Zhang et al., 2007) and that Hook1 is required for head–tail linkage in mice (Mendoza-Lujambio et al., 2002) (Table 1), it is possible that a more specialized KASH protein similar to ZYG-12 is yet to be identified that could bridge SUN5 and/or Spag4 to the centriole at the HTCA (Fig. 4A). This would be consistent with our model 1 and could explain why studies manipulating known classical KASH proteins like nesprins do not significantly impact male fertility. Furthermore, the role of ZYG-12 in the nucleus–centrosome linkage suggests that KASH domain-less isoforms of these proteins might be important in mediating connections between nuclei and centrioles. Given that previous studies have used mutations in the *Drosophila* KASH proteins Klar and Msp-300 that specifically remove the KASH domain (Kracklauer et al., 2010), more work is needed to test whether other regions of these proteins could still play a role at the HTCA.

Another specialized LINC complex appears to anchor centrosomes to the nucleus in the amoeba *Dictyostelium discoideum*. In this system, the SUN protein Sun1 occupies both the INM and ONM and is required for attachment of the centrosome and the nucleus (Schulz et al., 2009; Xiong et al., 2008). The proposed *Dictyostelium* KASH protein Interaptin does not appear to function in nucleus–centrosome linkage (Xiong et al., 2008). Sun1 is hypothesized to form a complex on both sides of the NE and interact with a perinuclear protein to facilitate this linkage (Graf et al., 2021). This is reminiscent of our model 3 (Fig. 4B), in which SUN proteins like Spag4 and SUN5 occupy the ONM to directly interface with cytoplasmic and centriole proteins. Alternatively, more similarly to model 1, it has been proposed that Kif9, a kinesin with a transmembrane domain that localizes to the ONM, might form a LINC complex with Sun1 to mediate nucleus–centrosome linkage (Graf et al., 2021; Tikhonenko et al., 2013), although an interaction between Kif9 and Sun1 has not been described.

*Dictyostelium* Sun1 shows further similarity to the HTCA SUN proteins. Like Spag4 and SUN5 in early spermatids at the developing manchette, *Dictyostelium* Sun1 is polarized on the side of the nuclear membrane facing towards the centrosome (Schulz et al., 2009). Overall, these and future studies of unique LINC complexes in model organisms could provide valuable insight into how the nucleus and centriole are attached at the HTCA.

## Conclusions

Our understanding of head–tail linkage at the HTCA in sperm is still limited. We propose that this linkage must be mediated by NE components, cytoplasmic components and centriole components. However, the precise molecular mechanisms by which these components function at the HTCA are largely unknown. It is important to note that sperm morphology is incredibly diverse; thus, it is possible that different molecular mechanisms at the HTCA evolved in different species. However, while the specific protein interactions involved might differ, we believe that the common theme of a head–tail linkage comprising NE proteins, cytoplasmic linkers and centriole proteins is likely conserved and serves as an important framework for understanding the molecular linkage at the HTCA.

There is a clear role for NE proteins in facilitating linkage at the HTCA, particularly testis-specific SUN-domain proteins like SUN5 and Spag4, whereas roles for other NE proteins like KASH proteins and NPCs remain underexplored. More work is necessary to understand how SUN proteins link to the centriole at the HTCA and whether this linkage is KASH protein dependent, NPC dependent or direct. Studying these processes in mammals is difficult due to limitations in mouse genetics and the lack of centriole structures in the mature sperm of many rodents. However, much can be learned from model systems like *Drosophila* that are amenable to targeted genetic experiments, high-throughput screens and live imaging. Another major hurdle is that most of our current knowledge comes from studies in which HTCA proteins were globally knocked out or knocked out during the entirety of sperm development, including during meiosis. This makes it difficult to study the roles of HTCA components, particularly centriole and cytoplasmic components, because many of these proteins are also essential during meiosis and for function outside of the testes. Thus, manipulation of HTCA proteins in specific stages of spermiogenesis in the testes will be necessary to determine the molecular linkage in the final HTCA structure. This could involve utilizing tools like deGradFP, which can directly deplete GFP-tagged proteins with spatial and temporal control (Caussinus et al., 2011, 2013), to specifically target proteins in spermatids at various stages. This technique has recently been used to successfully study the role of Nup358 at the manchette in spermatids (Li et al., 2023). Understanding how HTCA proteins function together at a molecular level is integral to understanding and combating male infertility that arises from HTCA defects. Furthermore, delineating this molecular linkage might offer potential targets for developing novel contraceptive options that target the male reproductive system, which are currently extremely limited.
